# Genetic Parameters of Age at Conception in Nellore Females Using Threshold Models

**DOI:** 10.1111/jbg.70029

**Published:** 2025-11-17

**Authors:** Raimundo Nonato Colares Camargo Júnior, Cláudio Vieira de Araújo, Marina de Nadai Bonin Gomes, Feliciano Benedetti de Freitas, Welligton Conceição da Silva, Simone Inoe de Araújo, Éder Bruno Rebelo da Silva, Alison Miranda Santos, José Ribamar Felipe Marques, José de Brito Lourenço‐Júnior, André Guimarães Maciel e Silva

**Affiliations:** ^1^ Postgraduate Program in Animal Science (PPGCAN), Institute of Veterinary Medicine, Federal University of Para (UFPA), Federal Rural University of the Amazon (UFRA), Brazilian Agricultural Research Corporation (EMBRAPA) Castanhal Brazil; ^2^ Department of Agricultural and Environmental Sciences Federal University of Mato Grosso (UFMT) Sinop Brazil; ^3^ Meat Technology and Processing Laboratory of the College of Veterinary Medicine and Animal Science, Universidade Federal do Mato Grosso do Sul Campo Grande Brazil

**Keywords:** bayesian, beef cattle, selection, variance, weaning, yearling

## Abstract

Age at conception is a critical factor in the intensification of production systems. It is crucial for intensifying production, optimising reproductive efficiency and boosting Nellore female productivity for genetic progress. However, the low heritability estimates currently limit the effectiveness of selection responses, thereby underscoring the necessity for more precise genetic parameter estimation methods. The aim of this study was to evaluate the genetic associations between body weights at weaning and yearling, and age at conception, in Nellore females. The methodology employed in this study involved the utilisation of a mixed polychotomous threshold model, a methodological framework that has been deemed appropriate for the analysis of categorical variables characterised by an underlying continuous distribution. The records of body weights at weaning and yearling, in conjunction with female age at conception, from 796 animals, were treated as a categorical variable associated with conception success. The (co)variance components were estimated via Bayesian inference using a Gibbs sampler. The mean heritability values were 0.43 (0.27; 0.60) for weaning weight, 0.63 (0.46; 0.81) for yearling weight, and 0.19 (0.06; 0.40) for the categorical variable of age at conception. While body weights exhibited a high additive genetic correlation were (0.79; 95% CI: 0.57; 0.96), correlations were lower between the categorical variable and weaning (−0.21; 95% CI: −0.75; 0.28) and yearling (0.34; 95% CI: −0.14; 0.71) weights. The study concluded that indicators of age at conception should incorporate additional selective criteria beyond body weight in order to improve the probability of conception.

## 1 Introduction

In order to enhance the technological level of livestock farming, it is imperative to utilise suitable animals that possess superior genetic merit (Yousuf et al. 2024). This objective is aimed at improving precocity, thereby enhancing reproductive efficiency and increasing productivity rates, including the pregnancy rate (Fernandes Júnior, Silva, et al. 2022).

In this regard, it is imperative to ascertain the genetic interrelationships between traits associated with productive and reproductive selection criteria (Burrow 2012). This knowledge facilitates the identification of the most suitable animal biotype for the specific conditions of commercial exploitation (Hozáková et al. 2020). In this sense, the estimation of genetic parameters without bias is of vital importance to guarantee the prediction of genetic values of animals (Da Silva Oliveira et al. 2025). Precise estimation of genetic parameters for traits such as sexual precocity in Nellore females is thus the essential basis for identifying superior genotypes and sustaining animal improvement programs aimed at overcoming the specific reproductive challenges of this herd, while also enabling the monitoring of genetic changes over time (Schmidt et al. 2021).

Improving the accuracy of genetic parameter estimation is a priority to ensure precision in genetic value prediction. This is essential to maximise the use of superior genotypes and increase productivity in beef cattle farming (Alves et al. 2021; Júnior et al. 2023).

In this context, the success of selection in a breeding program hinges on the adoption of a model that accurately represents the biological processes (Hickey et al. 2017). Moreover, it is imperative to comprehend the underlying sources of variations in phenotypic expression (Fodor et al. 2023). This knowledge is crucial for deriving estimates that minimise sampling error, thereby facilitating more precise predictions of genetic values (Silva Neto et al. 2024).

In summary, greater genetic progress in genetic improvement programs is achieved by defining the selection objective and adopting the most efficient criteria to achieve these objectives. In addition, more accurate identification of breeders is obtained through a more precise methodology in estimating the variance components associated with the selection criteria (Hofer 1998; Guo et al. 2025).

Some reproductive traits of interest are categorical in nature, requiring specific statistical approaches such as threshold models. Unlike continuous traits where standard linear models are directly applicable, the use of linear models for categorical variables can violate fundamental statistical assumptions, such as normality of residuals and homogeneity of variances. These violations can lead to biased and inefficient estimates of genetic parameters, thereby compromising the accuracy of genetic evaluations and selection decisions. However, the mode of inheritance of these traits is analogous to that of a trait with continuous variation. In such circumstances, the application of the threshold model in the estimation of variance components becomes a subject of interest (Marcondes et al. 2005; Martin Nieto et al. 2020).

In the context of analysing reproductive characteristics such as age at conception, which are inherently categorical, the use of threshold models is indisputable. This methodology, which allows genetic parameters for qualitative traits to be estimated accurately, was fundamentally developed by Gianola and Foulley (1983) and subsequently detailed and explored by Sorensen and Gianola (2002) in their book, which serves as a guide for its correct application in genetic improvement studies.

The principle of the threshold model for categorical traits was initially conceptualised by Wright (1934) and subsequently elaborated upon by Falconer (1965). This model posits that the phenotypic expression of a trait is associated with an underlying, unobservable continuous variable. This underlying continuous variable is assumed to be normally distributed, and the observed categorical outcomes are determined by a series of thresholds on this scale.

According to Hartl et al. (1997), categorical traits are classified as those with discontinuous phenotypes, in response categories, demarcated by the presence of thresholds on an underlying continuous scale of predisposition or risk. Collectively, these traits are referred to as quantitative traits, and, if not all, at least most of them are included in the realm of animal production (Mackay et al. 2009). Consequently, for traits such as age at conception, which, while reflecting a continuous biological process, are often recorded and analysed in discrete categories, the threshold model offers a statistically robust and biologically sound framework. It allows for the accurate estimation of genetic parameters by explicitly accounting for the categorical nature of the observed data while modelling the underlying continuous genetic predisposition, thereby providing more reliable insights for genetic selection compared to standard linear models.

Thus, the objective of this study was to estimate genetic parameters for body weights at weaning, at yearling, and for the age of the female at the time of conception. The latter was considered a categorical variable and was related to conception success in Nellore breed animals.

## 2 Materials and Methods

The dataset initially comprised 1232 records of body weights at weaning and yearling, as well as the age of females at conception, obtained from a Nellore cattle herd managed by Agropecuária Fogliatelli S/A, located at the Porto do Campo Farm, in the municipality of Lambari do Oeste, Mato Grosso, Brazil. The animals were maintained under a grazing system and received mineral supplementation in feed troughs. Pregnancy diagnosis was performed by rectal palpation, and only females that were diagnosed as pregnant and subsequently calved were considered for the analyses.

To ensure data quality and consistency, outliers were removed by excluding records located more than 3.0 standard deviations above or below the mean for body weights at weaning and yearling. Furthermore, sires with fewer than three progeny and contemporary groups with fewer than three weight records were excluded from the analysis. After data filtering and quality control procedures, a total of 796 valid records remained for statistical analyses.

The mixed linear model incorporated fixed effects of contemporary group, additive genetic random effects, and residual random effects. The Contemporary Groups (CG) in each measurement were defined with the inclusion of the year and month of measurement of the analysed body weight, considering CG with more than two animals with phenotypic information.

The age at conception was then classified into three categories: females with a positive diagnosis of conception up to 18 months of age received a score of 3, females with a positive diagnosis of conception over 18 months of age received a score of 2, and females that presented a negative pregnancy diagnosis received a score of 1.

For body weights at weaning and yearling, the age of the female at each weight was used as a covariate. The mixed linear model is represented by the following equation: Υ=Χβ+Ζα+ε, were Υ denote the vector of observations, and let β denote the vector of the set of fixed effects of contemporary groups and ages at each body weight (considered as covariates) associated with the incidence matrix Χ. Assuming the genetic values of the animals are represented by the vector a, the following relationship is established: $\alpha \sim Ν\left(0,A\sigma_{a}^{2}\right)$, where A and $\sigma_{a}^{2}$ is the matrix of the numerator of the coefficient of kinship between the animals and the additive genetic variance, respectively; ε the vector of residuals is assumed to be normally distributed and homoscedastic in variance, with a distribution given by: $e\sim N\left(0,I\sigma_{e}^{2}\right)$, where I and $\sigma_{e}^{2}$ are the identity matrix and residual variance, respectively.

In this sense, the random variables “weaning weight” and “yearling weight” were considered continuous, and the age at conception variable was considered categorical with three classes. Therefore, the threshold model was polychotomous, in which it assumed an underlying scale presenting a continuous normal distribution, as represented by the following equation: $U\left|\theta^{\prime }\sim N\left(W,I\sigma_{e}^{2}\right)\right.$, where U is the base scale vector; $\theta^{\prime }$ = ($\beta^{\prime },a^{\prime },c^{\prime }$) is the vector of location parameters; W is the incidence matrix; I is the identity matrix; and $\sigma_{e}^{2}$ is the residual variance.

The assumptions regarding the distribution of the vectors *Y*, *a*, and *e* in the mixed linear model are represented as: $\left[\begin{array}{c} y\\ a\\ e \end{array}\right]\sim \left\{\left[\begin{array}{c} \mathrm{X\beta}\\ \;\varphi \\ \;\varphi \end{array}\right],\left[\begin{array}{ccc} {\mathrm{ZGZ}}^{\prime }+R & \mathrm{ZG} & R\\ {\mathrm{GZ}}^{\prime } & G & \varphi \\ R & \varphi & R \end{array}\right]\right\}$, where *G* = *A* ⊗ *G* and *R* = *I* ⊗ *R*. Matrices *A* and *I* represent, respectively, the numerator relationship matrix and the identity matrix of order n, with n being the number of observations. *G*0 and *R*0 are the additive genetic and environmental covariance matrices, respectively.

The vector *a* ∼ *N*(0, *G*), where *G* = *A* ⊗ *G*0, and *G*0 follows an inverse‐Wishart prior distribution (conjugate for covariance matrices). The vector *e* ∼ *N*(0, *R*), where *R* = *I* ⊗ *R*0, and *R*0 also follows an inverse‐Wishart prior distribution. Additionally, the vector *β* ∼ *N* (*μβ*, ∑*β*), where *μβ* and ∑*β* are diffuse hyperparameters. Thus, *p*(*β*, *a*, *G*0, *R*0∣*y*) ∝ *p*(*y*∣*β*, *a*, *R*0)·*p*(*a*∣*G*0)·*p*(*β*)·*p*(*G*0)·*p*(*R*0).

The descriptive statistics of body weights at weaning and conception are presented in Table 1, while the frequencies between classes of the categorical variable are presented in Table 2.

**TABLE 1:** Number of records (*N*) means and standard deviations (SD) for body weights at weaning and yearling in females of the Nellore breed.

Variables	*N*	Body weights (kg)		Age (months)	
		Mean	SD	Mean	SD
Weaning	796	228.24	27.98	7.50	0.78
Yearling	796	411.19	75.64	23.21	6.09

**TABLE 2:** Absolute and percentage frequencies of the classes of the categorical variable referring to the age of conception, with means and standard deviations (SD) for the ages (months) of diagnosis in each class.

Classes	Frequency		Age at diagnosis (months)	
	Absolute	Percentage (%)	Mean	SD
1	388	48.74	21.85	4.72
2	237	29.77	30.46	2.27
3	171	21.48	20.71	3.19

*Note:* Class 1: Negative conception diagnosis. Class 2: Positive conception diagnosis over 18 months of age. Class 3: Positive conception diagnosis up to 18 months of age.

The (co)variance components were estimated by Gibbs sampling with the software Thrgibbs1f90 (Misztal et al. 2018). This process involved a chain of 2,000,000 cycles, with a burn‐in period of 500,000 cycles and a thinning interval of every 20 cycles. The Geweke diagnosis was utilised to assess the consistency of the chain size, employing a significance level of 0.05.

The genetic values of the animals in all the traits evaluated were predicted, and the Spearman correlation was obtained. This was done considering only breeders with more than 10 offspring in production and for all breeders with offspring in production.

## 3 Results

The posterior means of the additive and residual genetic (co)variance components for body weights at weaning (WW) and at yearling (WY), as well as for the categorical variable age at conception (AC), are shown in Table 3. These values are accompanied by the 95% posterior credibility interval and the posterior mean of the standard deviation.

**TABLE 3:** Monte Carlo errors, posterior means, 95% credible intervals (CI), standard deviations (SD) and effective sample size for additive genetic and residual (co)variance components of weaning weight (WW), yearling weight (WY), and the categorical trait age at conception (AC).

Genetics	Variance components					Effective sample size
	Monte Carlo error	Mean	CI (95%)		SD	
*Additive*						
WW	29.427	1201.00	799.00	1626.00	213.81	5279.2
WW, WY	54.759	429.91	259.80	613.80	90.30	371.9
WW, AC	0.4129	6.43	−2.65	15.44	4.47	317.4
WY	57.271	250.70	145.80	366.90	57.62	301.2
WY, AC	0.2146	−1.94	−6.34	2.38	2.21	305.8
AC	0.0174	0.32	0.06	0.64	0.16	288.1
*Residual*						
WW	22.298	683.69	364.10	994.80	161.45	5242.3
WW, WY	42.830	116.52	−15.82	257.80	69.88	366.2
WW, AC	0.3305	8.43	0.53	16.33	3.98	344.7
WY	47.861	330.68	236.50	426.70	48.88	304.3
WY, AC	0.1718	4.16	0.27	8.35	2.05	241.9
AC	0.0105	1.32	0.93	1.71	0.20	358.9

Table 3 presents the additive and residual (co)variance components, highlighting that the estimated additive variances for WW and WY were 1201.00 and 250.70, respectively. For AC, the additive variance was 0.32. These values, along with the residual variances, provide an initial insight into the magnitude of genetic variability present for each trait. Additive covariances between WW and WY (429.91), WW and AC (6.43), and WY and AC (−1.94) are also reported, serving as the basis for genetic correlations.

The analysis of the 95% confidence intervals (CI) presented in Table 3 is crucial to determine the statistical significance of the estimates of the (co)variance components. It was observed that the intervals for the additive (*𝜎* ^2^ *aᵢ*) and residual (*𝜎* ^2^ *eᵢ*) variance components of all traits (WW, WY, and AC) did not include zero, indicating that these estimates are statistically supported as different from zero. However, for some covariances, notably between weaning weight (WW) and age at conception (AC) (both additive and residual), and between weight at year (WY) and age at conception (AC) (also additive and residual), the 95% credibility intervals included zero. This suggests that these covariances are not statistically different from zero and, therefore, are not considered statistically supported by this study.

The effective sample size (ESS) values indicated that the MCMC chains generally exhibited good sampling efficiency, particularly for the main additive and residual variance components of weaning weight (WW), whose ESS values exceeded 5000, demonstrating excellent convergence and independence among samples. The Geweke diagnostic criterion also confirmed the satisfactory convergence of the Markov chains, reinforcing the reliability of the posterior estimates obtained for the genetic and residual components. In all subsequent analyses, probability levels greater than 0.05 were observed for the Geweke diagnostic criterion, indicating that the chain size, burn‐in, and thin‐in were sufficient for stationary chain sampling.

Consequently, the posterior means of heritability and genetic correlations between the evaluated traits were obtained (see Table 4).

**TABLE 4:** Posterior means (and 95% credibility intervals) for heritability (on the diagonal), additive genetic correlations (upper diagonal), and residual correlations (lower diagonal) among weaning weight (WW), yearling weight (YW), and the categorical trait age at conception (AC).

Variable	WW	WY	AC
WW	0.43 (0.27; 0.60)	0.79 (0.57; 0.96)	−0.21 (−0.75; 0.28)
WY	0.24 (0.05; 0,54)	0.63 (0.46; 0.81)	0.34 (−0.14; 0.71)
AC	0.28 (0.11; 0.42)	0.20 (0.03; 0.52)	0.19 (0.06; 0.40)

The heritabilities estimated in Table 4 indicate that yearling weight (YW; 0.63 [0.46; 0.81]) showed higher heritability than weaning weight (WW; 0.43 [0.27; 0.60]), suggesting that genetic effects become more pronounced at later stages of growth. The age at conception (AC; 0.19 [0.06; 0.40]) presented moderate heritability, implying that, although lower than for body weights, there remains sufficient additive genetic variance to allow response to selection. The additive genetic correlations revealed a strong positive association between WW and YW (0.79 [0.57; 0.96]) and a moderate positive correlation between YW and AC (0.34 [−0.14; 0.71]). In contrast, the correlation between WW and AC was weak and negative (−0.21 [−0.75; 0.28]), with wide credibility intervals suggesting low statistical support.

Spearman's correlations between predicted breeding values of sires for the evaluated traits followed a similar trend to that observed for the additive genetic correlations. Thus, sires tended to show an overall negative average for the predicted genetic values of weaning weight and a positive average for the other traits, with substantial re‐ranking observed particularly between weaning weight and the categorical trait (Table 5).

**TABLE 5:** Means with standard deviations (SD), medians, and Spearman correlations between predicted genetic values for weaning weight (WW), yearling weight (WY), and the categorical trait age at conception (AC) for sires with at least 10 offspring (above the diagonal, *n* = 28) and for all sires with progeny in production (below the diagonal, *N* = 82).

Criterion	Parameters			Spearman correlation		
	Mean	SD	Median	WW	WY	AC
WW	−5.27	10.85	−4.43	1.00	0.88	0.06
WY	29.55	29.61	30.57	0.77	1.00	0.45
AC	1.25	0.51	1.13	−0.01	0.57	1.00

The analysis of Spearman's correlations for the predicted genetic values of sires (Table 5) corroborates the trends observed in the additive genetic correlations. Notably, the correlation between WW and AC for sires with at least 10 offspring (0.06) and for all sires (−0.01) was negligible, indicating substantial re‐ranking of sires if age at conception is added as a selection criterion alongside weaning weight. This discrepancy in rankings, depending on the criterion and data quantity, emphasises the complexity in identifying superior animals for multiple traits and the need for comprehensive selection indices.

## 4 Discussion

The results indicate that the MCMC sampling process was effective in estimating the main variance components, providing robust and stable posterior distributions for the traits evaluated. The combination of satisfactory Geweke convergence statistics and adequate ESS values for the major traits supports the consistency of the Bayesian inference and the reliability of the estimated (co)variance structure (Table 3).

The posterior mean estimates of heritability for body weights (Table 4),0.43 (95% credible interval [CI]: 0.27; 0.60) for weaning weight (WW) and 0.63 (95% CI: 0.46; 0.81) for yearling weight (YW), indicate substantial additive genetic variability for growth traits in Nellore females. The relatively narrow credible intervals, especially for YW, indicate high precision of the estimates and reinforce confidence that additive genetic effects are the main determinant of the phenotype at both growth stages. The higher heritability observed for YW compared to WW likely reflects not only a reduction in maternal environmental influence, which tends to affect weaning weight, but also higher accuracy of phenotypic measurements at more advanced developmental stages. Therefore, YW emerges as a more reliable selection criterion for sustained genetic progress in growth performance. These results are consistent with previous studies reporting moderate to high heritabilities for growth traits in beef cattle (Utrera and Van Vleck 2004; Bolormaa et al. 2013; Fernandes Júnior, Peripolli, et al. 2022; Menezes et al. 2022; Rezende et al. 2022; Camargo‐Júnior et al. 2025), supporting the feasibility of achieving meaningful genetic gains through selection for these traits.

The posterior mean heritability for age at conception (AC; 0.19, 95% CI: 0.06; 0.40) indicates the presence of moderate additive genetic variance, suggesting that selection for improved reproductive precocity is feasible, albeit with slower expected progress than for growth traits. The relatively wide credible interval reflects greater uncertainty in the estimate, which is common for reproductive traits due to discrete or binary data and fewer observed events. Comparable results were reported by Schmidt et al. (2018), who found a heritability of 0.13 for age at first calving (AFC) and 0.19 for stayability (STAY), both closely related to reproductive efficiency. Other studies, however, have reported higher heritability estimates for reproductive precocity traits in Nellore cattle, such as heifer pregnancy (HP), with values of 0.42 (±0.02) by Santana et al. (2012) and 0.53 (±0.03) by Van Melis et al. (2010), or 0.45 (±0.02) for pregnancy at 16 months (HP16) by Boligon and Albuquerque (2011). Pereira et al. (2006) also reported heritabilities ranging from 0.51 to 0.76 for AFC using survival models. The wide variability of estimates in the literature, from 0.08 (Da Silva Neto et al. 2020) to 0.76 (Pereira et al. 2006), highlights the influence of trait definition, analytical model, and population structure on heritability estimation. Thus, while the present estimate indicates genetic potential, its interpretation should consider the magnitude and width of the CI, which signal greater uncertainty for AC relative to body weights.

The strong positive additive genetic correlation between WW and YW (0.79, 95% CI: 0.57; 0.96) confirms their close biological association and indicates that animals genetically superior for early growth tend to maintain performance at later stages. The relatively narrow credible interval reinforces confidence in the robustness of this relationship. In contrast, the moderate positive correlation between YW and AC (0.34, 95% CI: −0.14; 0.71) suggests that selection for higher yearling weight may favour animals that reach reproductive maturity earlier. However, the wide CI, including negative values, indicates that the direction and magnitude of this correlation are uncertain and may vary in different populations. Similar trends have been reported in studies linking growth and fertility traits (Carvalho Filho et al. 2022; Fernandes Júnior, Silva, et al. 2022; Brunes et al. 2024).

Conversely, the weak negative genetic correlation between WW and AC (−0.21, 95% CI: −0.75; 0.28), whose credible interval includes zero, reflects a possible antagonism between early growth and reproductive precocity, although the evidence is not statistically robust. This pattern is consistent with previous reports in Nellore cattle showing negative or null correlations between growth and reproductive traits (Boligon and Albuquerque 2011; Schmidt et al. 2018; Brunes et al. 2024). Such antagonistic trends may reflect physiological trade‐offs: animals allocating more energy to early somatic growth might delay reproductive development due to metabolic or endocrine limitations. From a breeding perspective, this observation highlights the importance of balanced selection strategies. Intensive selection for rapid growth at weaning could inadvertently delay reproductive maturity, ultimately reducing lifetime productivity in females (Costa et al. 2020).

It is important to emphasise that the phenotypic expression of WW, YW, and AC is determined not only by genetic factors but also by complex genotype × environment interactions. Variations in forage quality and availability, climatic conditions, disease incidence, and nutritional management can all influence the manifestation of genetic potential (Menezes et al. 2022; Fodor et al. 2023). For instance, nutritional restriction can obscure genetic differences in growth, leading to underestimation of genetic merit, whereas harsh environmental conditions may delay sexual maturity independently of genotype (Costa et al. 2020). Therefore, accounting for environmental heterogeneity and *G* × *E* interactions is essential for accurate estimation of genetic parameters and for the design of sustainable selection programs that integrate growth performance and reproductive efficiency.

Although the definition of CG helps control part of this non‐genetic variance, it may not capture the totality of environmental and management heterogeneity. The significant presence of *G* × *E* interactions is particularly relevant in tropical production systems, such as the one in this study, where environmental variations are pronounced (Burrow 2012; Camargo‐Júnior et al. 2025; Carvalho Filho et al. 2022; Silva Neto et al. 2024). When *G* × *E* is substantial, the ranking of superior animals can change dramatically between different environments, indicating that a genotype that performs well in one scenario may not maintain that performance in another. This suggests that low correlation values and the lack of statistical support for some genetic relationships, such as between WW and AC, may partly reflect the action of environmental and management factors that attenuate or alter genetic expressions. Consequently, to optimise genetic progress, it is essential to develop selection strategies that not only consider multiple criteria but are also solid in the face of environmental and management variations, aiming to identify animals with consistent performance in different contexts.

These findings highlight the importance of multi‐trait selection strategies that simultaneously target growth and reproductive performance. For instance, selecting solely for weaning weight (WW) may unintentionally negatively impact reproductive efficiency, whereas incorporating yearling weight (WY) and scrotal circumference (SC) into selection indices could promote a more balanced genetic response, enhancing both productivity and fertility. However, these genetic correlations should be carefully evaluated based on production objectives—whether the primary focus is early‐market weight, maternal efficiency, or reproductive turnover rate.

The Spearman correlations among predicted breeding values (Table 5) provide valuable information about sire ranking consistency across different traits. The strong genetic associations between WW and WY (0.88 and 0.77 for different sire groups) confirm the expected patterns of genetic correlation, while the negligible correlations between WW and SC (0.06 and −0.01) indicate significant re‐ranking of sires when different selection criteria are applied. These ranking variations underscore the necessity of using comprehensive selection indices that properly account for the relative importance of each trait within the breeding program. Exclusive focus on growth traits in sire selection may compromise genetic improvement of reproductive traits, particularly in production systems where female fertility is paramount.

The distribution of predicted breeding values reveals an interesting genetic pattern, with some sires showing negative values for WW but positive values for both WY and SC. This suggests that certain sires, while potentially unfavourable for early growth, may carry beneficial alleles for later development and reproductive traits. Such genetic complexity highlights the need for advanced selection methodologies that can identify sires offering balanced genetic contributions across multiple economically important traits. This reinforces the importance of multi‐trait selection indices that balance productivity and reproductive efficiency (Fernandes et al. 2021).

Despite the relevance of the results of this study for understanding the genetics of age at conception in Nellore females, it is essential to highlight some limitations that may influence the generalisation and accuracy of the results obtained. The first limitation lies in the size of the dataset, which included 796 animals. Although it was sufficient for the detection of moderate heritabilities, a larger number of records could have provided estimates of genetic parameters with greater precision and narrower credibility intervals, which is particularly relevant for low‐magnitude correlations. Second, there is a marked imbalance in the classes of the categorical variable age at conception (Table 2, 48.74% negative diagnoses and 21.48% early conceptions). Although threshold models are designed to handle unbalanced categorical data, the disparity in class frequencies may have impacted the model's ability to fully discriminate between thresholds, especially for the less frequent class, possibly affecting the accuracy of estimates for precocity. Finally, while the use of contemporary groups controlled for important management and environmental variations, it is plausible that the absence of more detailed environmental covariates, such as daily climate data, pasture quality indices, information on parasite load, or specific nutritional programs, left part of the environmental variance unmodeled. These factors, when uncontrolled, may overlap or interact with genetic effects (*G* × *E*), leading to estimates of heritability and correlations that may not fully reflect biological complexity across a broader spectrum of conditions. Future studies with more extensive datasets and the inclusion of more specific environmental covariates could further refine the understanding of these relationships.

## 5 Conclusions

The presence of additive genetic variability in the evaluated traits is sufficient to promote genetic progress, particularly with regard to body weights.

A strong correlation was not observed between body weights and the categorical variable. This finding indicates a need to explore the association of these variables with other criteria that are more closely related to successful conception.

Despite its contributions, this study was limited by a moderate dataset size and imbalance in age classes at conception, which may have impacted the accuracy of estimates. Future research with more comprehensive data and detailed environmental covariates may refine our understanding of these relationships.

## Author Contributions

Experiment design: R.N.C.C.J., C.V.A., J.R.F.M. and A.G.M.S. Experiment execution: R.N.C.C.J., M.N.B.G., W.C.S., C.V.A., F.B.F., S.I.A., J.R.F.M., S.I.A., A.M.S., J.B.L.‐J. and A.G.M.S. Investigation: A.S.O., M.N.B.G., R.N.C.C.J., W.C.S., C.V.A., A.M.S., J.R.F.M., S.I.A., and J.B.L.‐J. Data curation: R.N.C.C.J. and C.V.A. Formal analysis: M.N.B.G., A.G.M.S., and C.V.A. Original writing: R.N.C.C.J., C.V.A., and A.G.M.S. All authors have read and agreed to the published version of the manuscript.

## Consent

The authors have nothing to report.

## Conflicts of Interest

The authors declare no conflicts of interest.

## Data Availability

The data presented in this study are available upon reasonable request from the corresponding author.
